# Re-Appearing Legacy Effect in Timing of Autumnal Leaf Senescence and Compensation Growth After Severe Drought in *Fagus sylvatica* L.

**DOI:** 10.3390/plants15182867

**Published:** 2026-09-19

**Authors:** Kristine Vander Mijnsbrugge, Sofie Vanneste, Sharmila Majumder, Marc Schouppe, Stefaan Moreels, Sharon Moreels, Simeon Beeckman

**Affiliations:** Department for Forest Ecology and Management, Research Institute for Nature and Forest (INBO), 9500 Geraardsbergen, Belgium; sofie.vanneste@gmail.com (S.V.); sharmilamou.126@gmail.com (S.M.); marc.schouppe@inbo.be (M.S.); stefaan.moreels@inbo.be (S.M.); sharon.moreels@inbo.be (S.M.); simeon.beeckman@gmail.com (S.B.)

**Keywords:** latent legacy effect, European beech, water withholding, compensation growth, bud burst, autumn leaf senescence, leaf chlorophyll content, phenological memory, post-drought recovery

## Abstract

Drought events are expected to induce both immediate and delayed effects on tree functioning, yet the persistence and dynamics of these legacy effects remain poorly understood. In an experimental set-up, European beech (*Fagus sylvatica* L.) saplings were subjected to two consecutive water-withholding treatments during the 2022 growing season, resulting in five drought-severity categories. We monitored these categories over the following three years to assess drought legacy effects on phenology and growth. In the first post-drought year, bud burst proceeded more slowly in all drought categories, with the plants exposed to severe summer drought displaying the longest duration. Minor differences between drought categories and control plants were observed in timing of autumn leaf senescence in the first post-drought year and in the following bud burst, suggesting recovery in these phenological responses. Unexpectedly, differences re-emerged in autumn leaf senescence in the second post-drought year, when all drought categories displayed delayed senescence. This differentiation persisted into the following spring, with delayed bud burst in the severe spring- and summer-drought categories. The severe spring-drought categories maintained delayed senescence in the third post-drought year. Diameter and height growth were strongly reduced in the first year following drought. Although growth differences largely disappeared in the second post-drought year, the severe summer-drought categories exhibited enhanced diameter growth in the third year, indicating compensatory growth. Together, these results demonstrate that drought legacy effects in *F. sylvatica* are dynamically expressed over multiple years, and that such temporal variability complicates predictions of long-term forest responses to increasingly frequent episodic drought events under climate change.

## 1. Introduction

Climate change is predicted to increase the frequency, duration, and intensity of drought events across Europe, representing a major threat to the functioning and resilience of temperate forests [1,2]. Climate projections suggest that extreme droughts will become more frequent under ongoing warming, intensifying water limitation during the growing season and exposing forest ecosystems to unprecedented stress (Intergovernmental Panel on Climate Change) [3]. In woody plants, drought induces a cascade of physiological responses, beginning with stomatal closure and reduced photosynthesis and progressing towards increased risk of hydraulic failure, decreased carbon assimilation, depletion of non-structural carbohydrate reserves, embolism, vascular damage and ultimately tree death [4,5,6,7]. Accordingly, drought events are consistently associated with reduced tree growth [8,9]. Tree-ring studies have documented pronounced growth reductions in trees following severe drought events, with legacy effects persisting for several years [10,11]. In natural forest conditions, drought responses are further shaped by stand structure, species mixtures, soil properties and microclimate [12,13,14,15,16]. Altered physiology and reduced radial growth are typical legacy effects of drought in temperate tree species, but the magnitude and duration can vary strongly with species, drought severity and environment and site conditions [17,18]. The legacy effects are thought to be driven by underlying stress-memory mechanisms operating at the physiological and molecular level. Epigenomic modifications, including changes in DNA methylation, histone marks, and regulatory small RNAs, have been proposed as key mechanisms underlying drought stress memory in plants [19,20].

The timing of the leaf phenophase bud burst and autumn senescence are generally positively correlated [21,22], a phenomenon that has been attributed to the leaf longevity constraint, which limits variation in the time span between bud burst and leaf senescence [23]. Drought during the growing season can disturb this correlation, often by advancing autumn senescence [24,25]. In a rain exclusion experiment on a young beech plantation, drought advanced leaf senescence (and delayed the subsequent bud burst) [26]. Earlier leaf senescence shortens the growing season and may, in severe cases, increase the risk on tree mortality [27]. However, while less severe drought during the growing season can advance autumn leaf senescence, a delay of this phenophase can be triggered by severe drought; these two contrasting responses have been observed in similar experiments for *Fagus sylvatica* [28], *Prunus spinosa* [29] and *Cornus sanguinea* [30].

Water deficits suppress cambial activity and cellular differentiation in the stems of woody plants, making moisture availability the principal limiting factor for stem diameter increment [31]. The timing of a drought period in the growing season can influence the effects on tree growth, with species that concentrate height or radial growth in spring being particularly sensitive to spring drought [32]. Interestingly, in their study on potted tree seedlings, van Kampen et al. [32] found that for one out of six of the studied tree species, *Thuja occidentalis*, a spring drought not only reduced growth during the drought but also led to enhanced growth after the drought had ended.

*F. sylvatica* is a dominant and economically highly valued forest tree species in many areas in Europe. Various modelling studies attempt to project how the geographic distribution of the European beech (*F. sylvatica*) will shift across Europe under different climate change scenarios [33,34]. The species has received particular attention in drought studies as it is considered relatively sensitive to drought due to its shallow rooting system and comparably vulnerable hydraulic system as reviewed by [35]. Controlled experiments with potted saplings provide valuable insights into the mechanistic basis of drought responses and their carry-over effects. As these experiments are typically performed in uniform environmental conditions, the disentanglement of direct drought effects from confounding environmental variability is facilitated [36,37]. The present study investigates the legacy effects of an experimental drought treatment on potted beech saplings, with a particular focus on leaf phenology (bud burst and leaf senescence) and growth (diameter and height increment). We hypothesised that drought effects would clearly influence the leaf phenophases and growth variables up to one post-drought year and would gradually diminish thereafter. This work aims to improve our understanding of how drought exposure can shape the subsequent performance of beech.

## 2. Results

**Figure 1:**
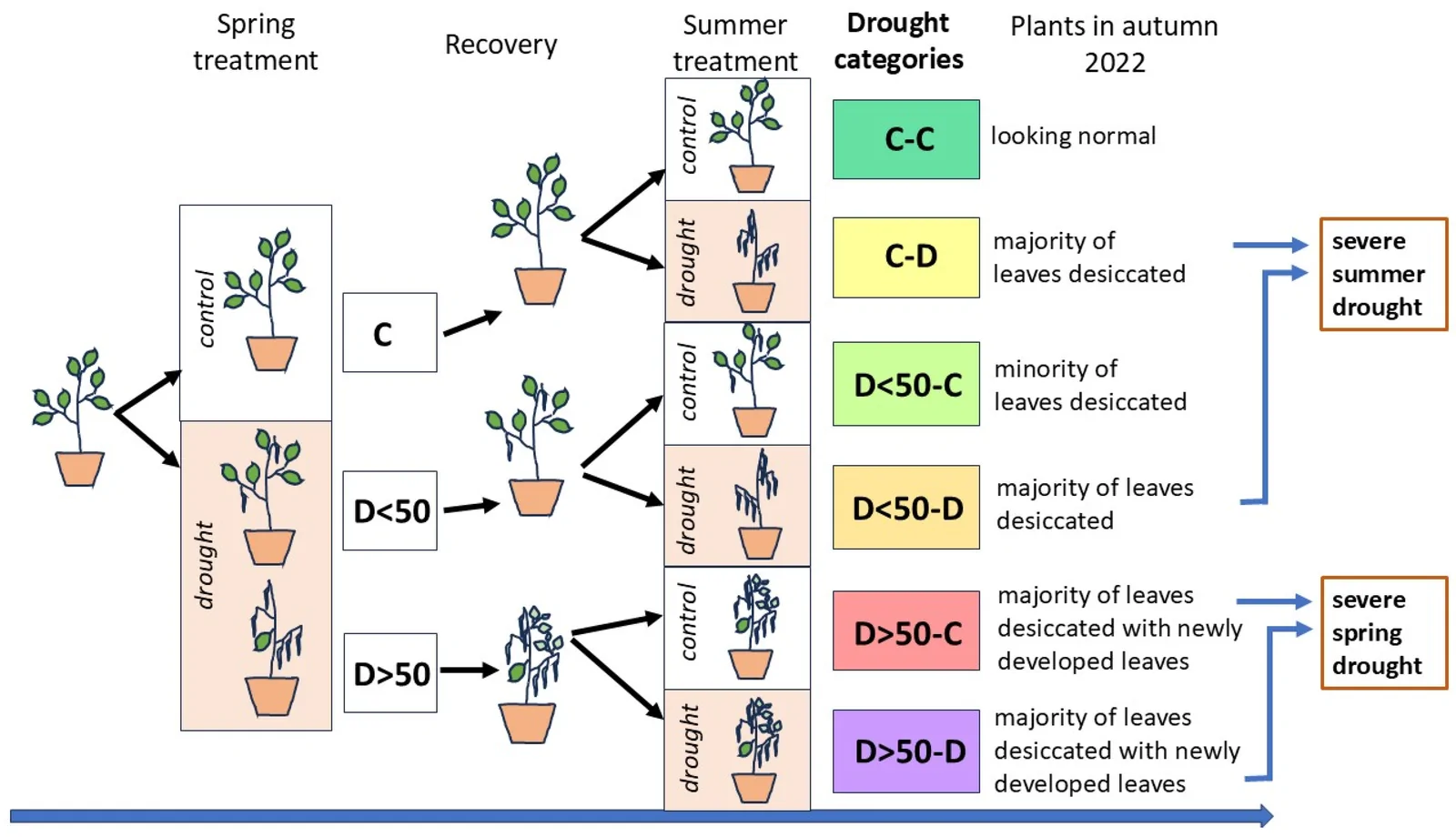
Schematic representation of the two subsequent drought treatments in 2022. C: Control; D: drought treatment; D<50 and D>50: less or more than 50% of the leaves desiccated in the spring-drought treatment. Based on the timing of the drought and the severity of the developed symptoms, saplings were grouped in 6 drought categories. Visually, there was no distinction between the drought symptoms of categories D>50-C and D>50-D at the end of the summer treatment.

Leaf phenology and growth in the years after the water-withholding treatment of 2022 were influenced by the drought exposure, which is described in the Materials and Methods Section and visualised in Figure 1.

### 2.1. Leaf Phenology and Chlorophyll Content Index

**Figure 2:**
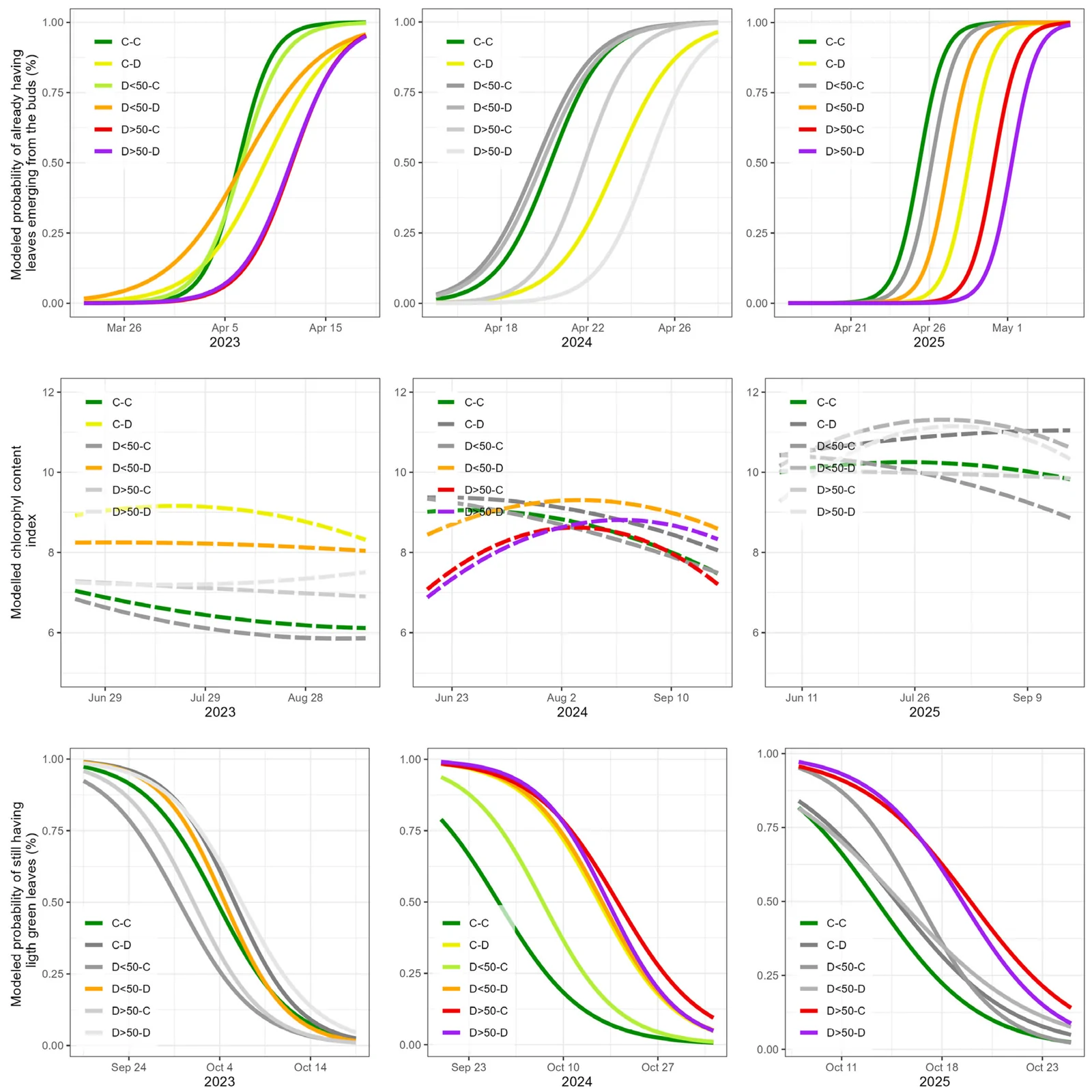
Modelled bud burst, leaf chlorophyll content index and autumn leaf senescence for the double controls (C-C) and the different drought categories in the three post-drought years 2023 till 2025. The two phenophases were modelled using cumulative logistic regression, whereas chlorophyll content index was modelled applying linear regression. Drought categories are in Figure 1. Drought categories significantly differing from the double controls are indicated in colour; not significantly differing drought categories are indicated in grey.

In the spring of 2023, bud burst was clearly affected by the drought treatments in the previous year (Figure 2 and Figure S1). All drought categories were characterised by a significant interaction term between day and treatment (Table S1), signifying a slower bud burst process compared to the double controls. Based on the model statistics, we calculated the number of days required for plants in each drought category to develop from winter buds to unfolded leaves. The duration of the bud burst process for the category of saplings that developed mild symptoms during the spring-drought treatment in 2022 and that displayed an earlier autumn leaf senescence in this year (19.5 days) was closest to the double controls (15.5 days). Saplings that developed severe symptoms during the spring drought (D>50-C and D>50-D) resprouted during the post-drought recovery and both categories were characterised by a slower bud burst process (22.9 and 23.7 days, respectively). Saplings that developed severe leaf desiccation in the summer drought (C-D and D<50-D) did not resprout in the post-drought recovery and they displayed the largest duration of bud burst in 2023 (31.5 and 36.5 days, respectively). The slowest bud burst of the severe summer-drought categories (C-D and D<50-D) was followed by a higher chlorophyll content index (9.3 ± 4.4 and 8.3 ± 3.5, respectively) compared to the double controls (5.9 ± 1.6) during the 2023 growing season (Table S2, Figure 2 and Figure S2).

Although the timing of autumn leaf senescence in 2022 differed among drought categories, with one category senescing earlier and the others later than the double controls, autumn leaf senescence in 2023 showed hardly any significant differences between the double controls and the different drought categories (one significant interaction term D<50-D:Day with a *p*-value = 0.044 in Table S1, Figure 2 and Figure S1).

In the spring of 2024, two years after the drought treatment, again, hardly any significant differences were present between the double controls and the different drought categories (one significant interaction term C-D: Day with a *p*-value = 0.02 in Table S1, Figure 2 and Figure S1). During the growing season of 2024, several drought categories displayed chlorophyll content index trajectories that differed from the double controls (Figure 2 and Figure S2, Table S2). Three out of four severe drought categories (D<50-D, D>50-C and D>50-D) reached their maximum values later, while no drought categories clearly displayed higher values than the double controls at all three time points.

In the following autumn, all the drought categories displayed a significantly delayed leaf senescence relative to the double controls, with the category representing the minor leaf desiccation (D<50-C) being less delayed (8 days) than the other categories (Figure 2 and Figure S1, Table S1). The two severe spring droughts D>50-C and D>50-D were delayed with 21.5 and 19.7 days, respectively, and the two severe summer droughts C-D and D<50-D were delayed with 18.2 and 18.7 days, respectively.

In the spring of 2025, three years after the drought treatment, bud burst was delayed in three severe drought categories relative to the double control (C-D, D>50-C and D>50-D, with delays of 3.1, 4.8 and 5.9 days, respectively) (Figure 2 and Figure S1, Table S1). In the 2025 growing season, leaf chlorophyll content index no longer differed among the drought categories (Figure 2 and Figure S2, Table S2), whereas in the autumn, leaf senescence was still delayed for the severe spring-drought categories (6.5 and 5.9 days for D>50-C and D>50-D, respectively) (Figure 2 and Figure S1, Table S1).

**Figure 3:**
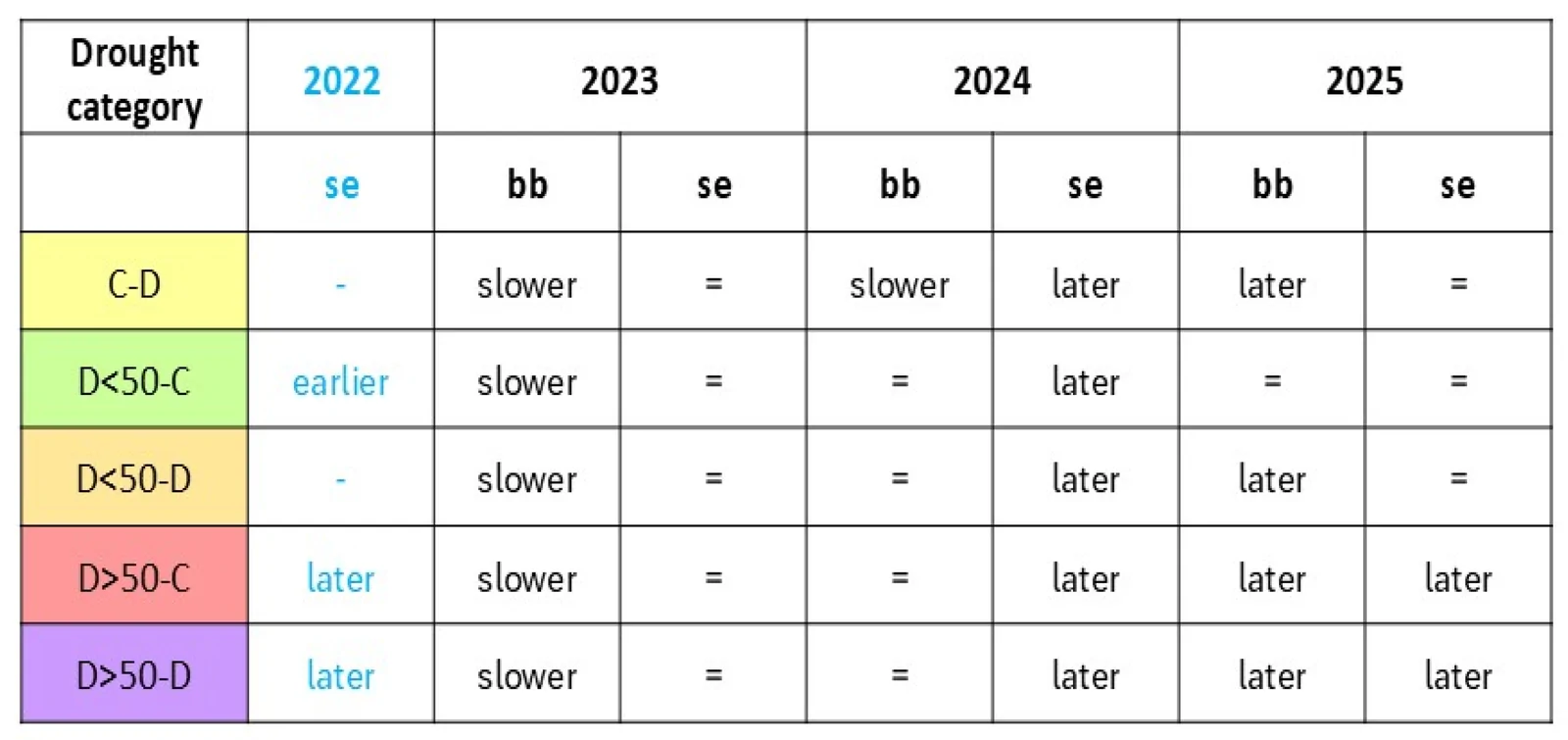
Schematic representation of the phenological responses in the post-drought years 2023 till 2025. The different drought categories were compared to the double controls (C-C). The responses in the year of the drought (2022) were published previously and are indicated in blue. =: No significant difference with C-C; -: no foliage thus no leaf senescence scoring; bb: spring bud burst; se: autumn leaf senescence. Drought categories are summarised in Figure 1.

The phenological responses in the three post-drought years are summarised in Figure 3.

### 2.2. Growth

**Figure 4:**
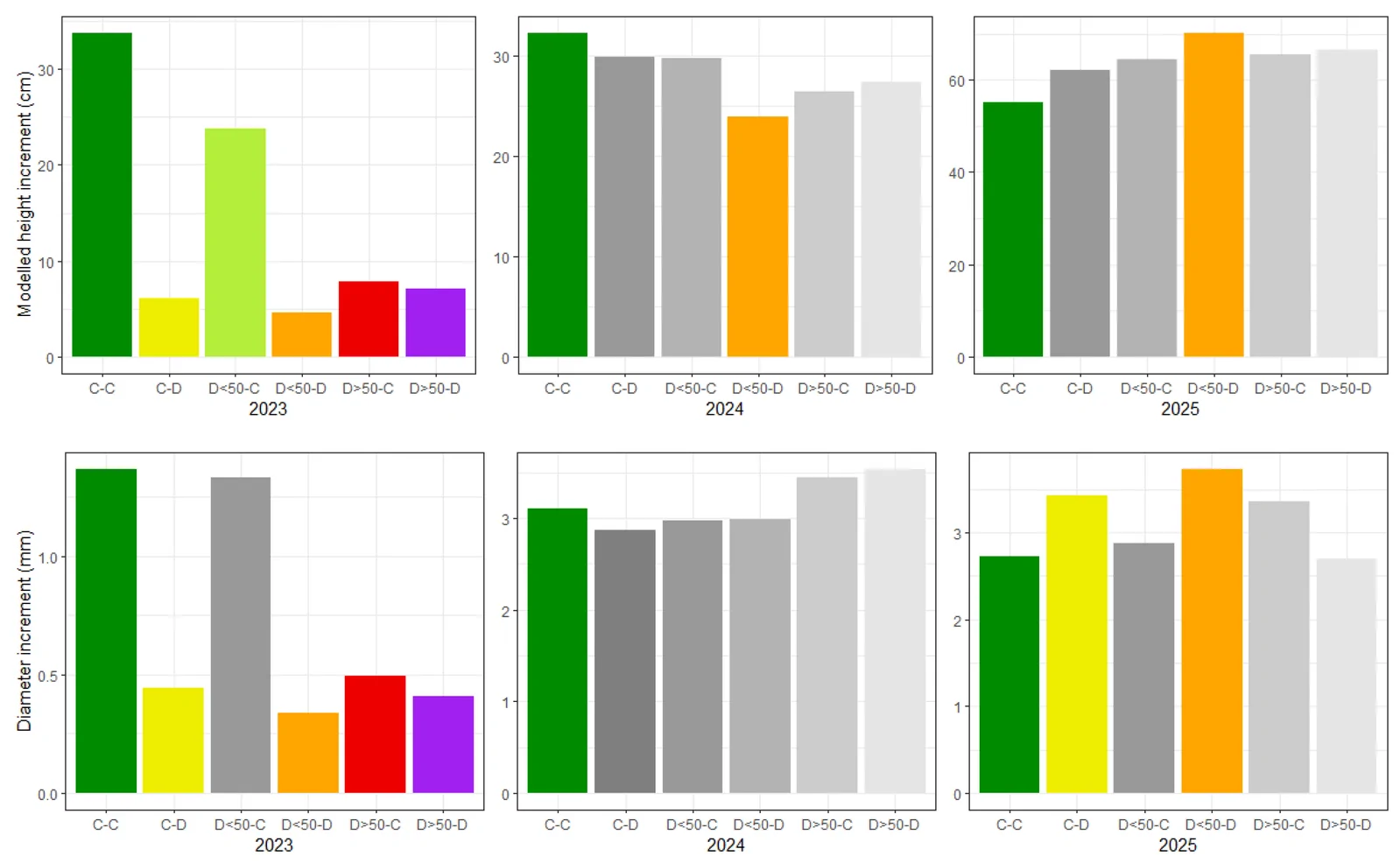
Modelled height and diameter increment for the controls (C-C) and the different drought categories of 2023 till 2025. The double controls are the standard to which the different drought categories are compared to. Drought categories are summarised in Figure 1. Drought categories significantly differing from the double controls are indicated in colour; not significantly differing drought categories are indicated in grey.

In 2023, strong negative effects of the drought stress experienced in the preceding year were observed for both height and radial increment in all the severe spring- and summer-drought categories (D>50-C, D>50-D, C-D and D<50-D) (Table S3, Figure 4 and Figure S3). The category with mild stress symptoms (D<50-C) showed a significant although relatively modest reduction in height increment (23.6 cm ± 12.4 cm compared to 33.1 cm ± 11.5 cm for the double controls) and no reduction in radial increment.

In 2024, two years after the drought treatments, increments in both height and diameter in nearly all categories did not differ significantly from the double controls. Only the D<50-D category grew a little less in height than the double controls in this year.

Three years after the drought treatments, in 2025, several drought categories showed significantly higher values than the double controls. For height, the D<50-D category showed a significantly higher increment (69.5 cm ± 32.3 cm compared to 56.3 cm ± 22.5 cm for the double controls). For the radial growth in this year (2025), the two severe summer-drought categories (C-D and D<50-D) were characterised by a diameter increment being significantly higher than the double controls (3.50 mm ± 1.93 mm and 3.87 mm ± 1.81 mm, respectively, compared to 2.64 mm ± 2.02 mm for the double controls).

**Figure 5:**
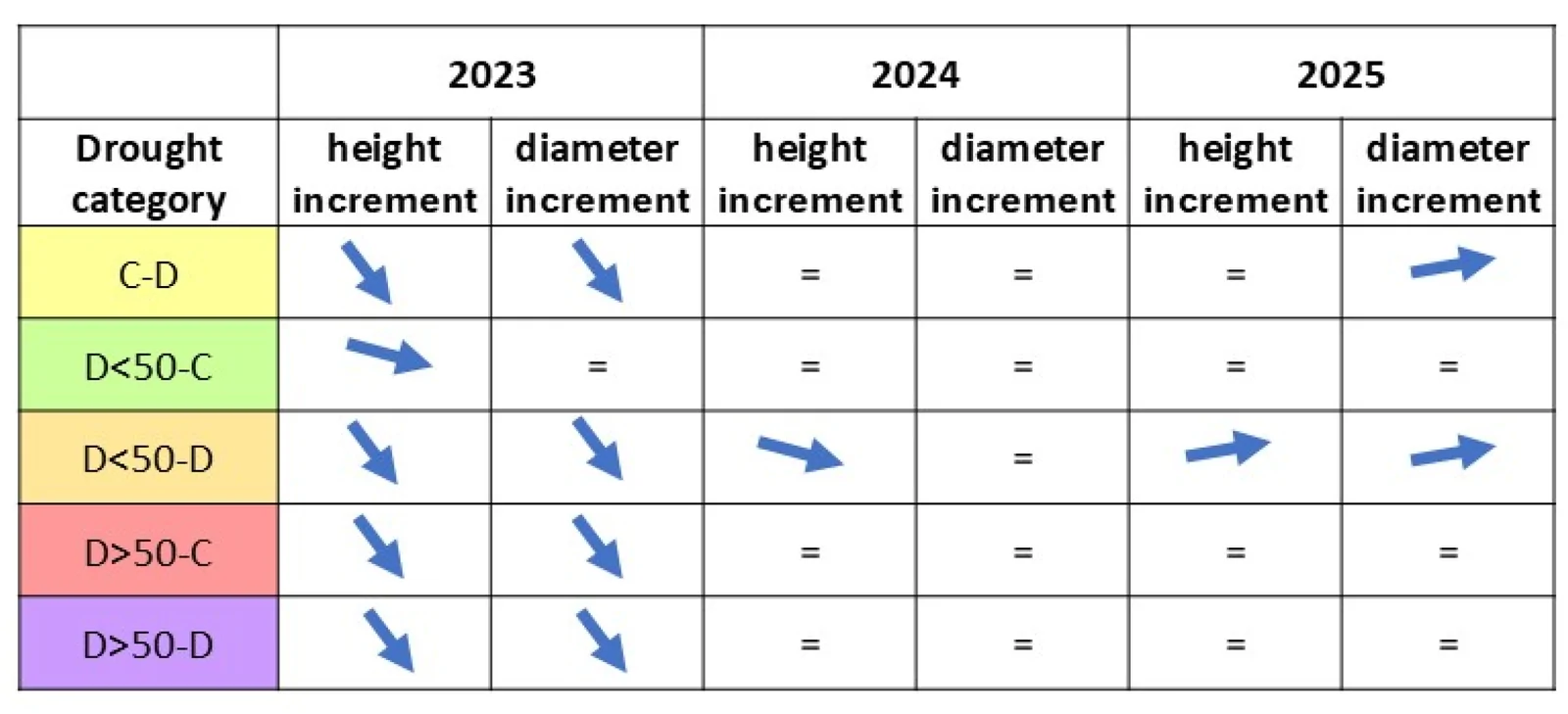
Schematic representation of the growth responses in the post-drought years 2023 till 2025. The different drought categories are compared to the double controls (C-C). =: No significant difference from C-C. Arrows indicate less (downwards) or more (upwards) increment growth. Drought categories are summarised in Figure 1.

The growth responses in the three post-drought years are summarised in Figure 5.

## 3. Discussion

### 3.1. Phenological Legacy Effects

In the year of the drought treatments (2022), the category with mild symptoms in the spring drought showed an advanced autumn leaf senescence, the severe spring-drought categories (D>50-C and D>50-D) resprouted after rewatering and the foliage stayed longer green into autumn, whereas the severe summer-drought categories (C-D and D<50-D) did not resprout after rewatering [28]. In spring 2023, bud burst developed more slowly in all drought categories compared to the double controls, with the mild symptoms category in the spring drought showing a relatively small increase in duration, the severe spring-drought categories an intermediate increase and the severe summer-drought categories showing the longest duration. These results corroborate the reported finding that in *F. sylvatica*, drought-induced reductions in carbon availability are linked with weaker buds [38]. The variable increase in bud burst duration among the different drought categories suggests that longer bud burst durations are indicative of more severe stress conditions during the drought. This interpretation is consistent with the higher chlorophyll content index during the growing season of 2023 in the categories with longest bud burst duration (severe summer drought).

As leaf senescence in 2023 and the following bud burst in 2024 hardly expressed the drought categories, it was tempting to conclude that the effect of the drought treatments in 2022 on leaf phenology had faded away by early 2024. Strikingly, however, the timing of autumn leaf senescence two years after the drought treatments separated all the drought categories significantly from the double controls. Whereas the mild-drought category had shown advanced senescence in the drought year and no difference in timing of leaf senescence in 2023, it displayed delayed senescence in 2024. This may signify a shift from an avoidance strategy, in which the plant protects itself to putative new drought episodes by an earlier leaf senescence, towards a more tolerant strategy in which the plant maintains normal growth while still prolonging its autumnal photosynthetic activity as recovery from the experienced stress [39]. The more severe drought categories that were already delayed in 2022 were again delayed in 2024. In the year of the drought treatments, the delay of autumn senescence was interpreted as a strategy to extend carbon gain through photosynthesis, replenish depleted reserves, and repair damaged tissues [40]. Because this strategy may increase the risk of early-autumn frost injury, it may reflect the severity of drought-induced impairment [30,41].

**Figure 6:**
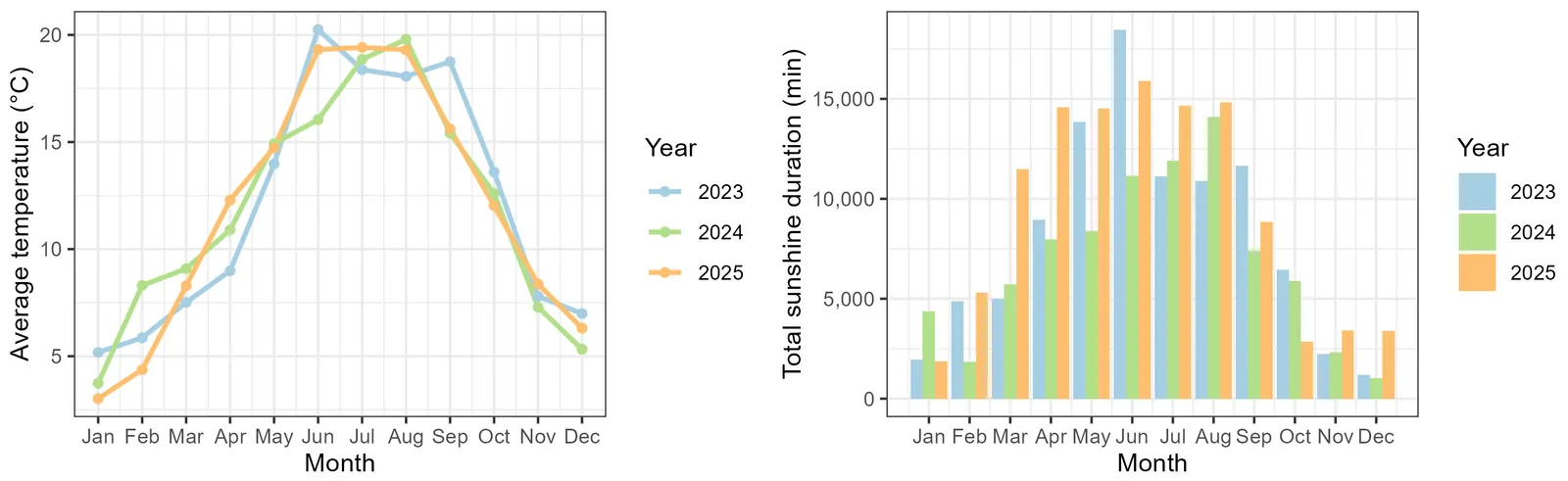
Mean monthly temperature and monthly sunshine duration for the three studied post-drought years, 2023 till 2025, in Uccle, Belgium (50 km distance from container field in Geraardsbergen, Belgium). Data source: Royal Meteorological Institute of Belgium, Open Data platform [44].

The re-emergence of drought legacy effects in autumn senescence, after phenological differences had temporarily faded and height and radial growth had recovered, remains difficult to explain. It can be hypothesised that drought legacy effects in the leaf phenophases of beech last several years and become more clearly expressed under favourable growing conditions while being masked in a more stressful environment. In our experiment, this would signify that 2023 was a more stressful year for the saplings compared to 2024. As European beech is a shade-tolerant species [42], it may be vulnerable to elevated solar radiation and the associated heat stress, particularly when drought limits transpirational cooling [43]. During the growing season (April to September), the saplings experienced on average 15% more solar radiation in 2023 compared to 2024 (Figure 6). Specifically, May and June were characterised by higher solar radiation and June by higher temperatures in 2023 (Figure 6).

We hypothesise that the legacy effects that we observed may represent a longer-term stress-memory mechanism that can be associated with epigenetic modifications [19,45]. The weaker expression of stress memory under more adverse growing conditions may be related to the finding that population differentiation in growth traits (genetic effects) in provenance trials planted on favourable sites is enhanced, whereas on harsher sites the growth differences among provenances are minimised [46,47]. In a common garden of Prunus spinosa, the population differentiation in autumn leaf senescence due to different climates at the home sites of the provenances (genetic effects) was likewise reduced under drought stress [29]. In our experiment, the delayed leaf senescence for the mild-drought category in 2024, while being advanced in the year of the drought (2022), suggests that the longer-term stress memory may favour extended carbon gain during autumn above protection against putative new drought events by an earlier senescence. In addition to this hypothesis, resource allocation dynamics may have contributed to the observed effects. Notwithstanding the restored (normal) pattern in height and radial growth in 2023, some physiological processes may not have been fully restored in this year, such as resources of non-structural carbohydrates, vascular repair or root-to-shoot signalling. These putative aspects may have contributed to the re-appearance of the differentiation in leaf senescence timing of the drought categories in 2024.

### 3.2. Growth Responses

Drought reduces growth by limiting photosynthesis [35] and severe drought increases the post-drought carbon cost required to restore normal functionality in woody plants [48]. In our experiment, as expected, growth in the year after the drought treatment was strongly diminished for all severe drought categories, with no clear distinction between the spring (D>50-C and D>50-D)- and the summer-drought categories (C-D and D<50-D). Nevertheless, the severe summer-drought plants (C-D and D<50-D) that did not resprout after the drought treatment in 2022 showed a higher chlorophyll content index than the controls during the growing season of 2023, which was not the case for the severe spring-drought categories (that did display post-drought resprouting in 2022). Post-drought recovery has previously been associated with higher photosynthetic activity in *F. sylvatica*, interpreted as a need to replenish carbohydrate reserves and support regeneration and growth [49,50]. A post-drought growth reduction concurring with an elevated photosynthetic activity can be explained as carbon resources being allocated away from radial growth and toward recovery processes [18]. Our results support the uncoupling of growth and photosynthesis in the post-drought year, with the higher chlorophyll content index in the year after the drought being related to the timing of the drought event in the year of the drought, because this timing determined whether plants resprouted in the drought year itself or not. Likely, for the spring-drought plants, reserve replenishment was already taking a start after resprouting and during the delayed leaf senescence in the autumn of the drought year, whereas it only took place in the year after the drought treatment for the summer-drought plants, which could explain why only the summer-drought plants displayed a higher chlorophyll content index in the post-drought year.

Two years after the drought, radial and height growth returned to the level of the control plants for most drought categories, consistent with reports that beech growth can be strongly reduced by drought but can also recover relatively quickly [9,51]. Similar as in the first year after the drought, the chlorophyll content index during the growing season was higher for the summer-drought category D<50-D. Surprisingly, in this second year, the chlorophyll content index was also higher for the spring-drought categories (D>50-C and D>50-D), which had not differed from the controls in the first year after the drought. This elevated chlorophyll content index in the second year may be linked to the same stress-memory process that also underlays the delayed senescence observed in all drought categories this year.

Three years after the drought we observed a growth pattern consistent with compensation growth, a phenomenon defined as accelerated growth following a period of resource limitation or stress [52] and which has been widely observed across organisms including woody species [53,54,55,56]. For European beech, for instance, compensation growth has already been described after heat stress [57]. Our results again indicate a separation between spring-drought plants, which recovered by resprouting immediately after the drought, and summer-drought plants, which did not resprout, because only the summer-drought categories (C-D and D<50-D) showed compensatory radial growth. For height growth, only the category D<50-D exceeded the controls. These findings suggest that the stress memory imprinted in the year of the drought depended not only on the severity of the experienced stress, as the mild stress category did not display compensation growth, but also on the timing of it in the growing season. This observation can be related to compensatory growth in grassland research, with grasslands subjected to summer drought tending to become primed for greater compensatory growth in the following year when compared to grasslands experiencing drought earlier in the growing season [58].

## 4. Materials and Methods

### 4.1. Experimental Set-Up

The drought experiment was conducted in 2022 on 281 potted *Fagus sylvatica* saplings, as described previously [28]. In short, saplings originating from seeds collected in 2016 were germinated in 2017 and subsequently maintained in containers in a common garden setting. A drought experiment was performed during the 2022 growing season, consisting of two consecutive water-withholding treatments (factorial design): in spring (23 May–15 June) and in summer (8 August–12 September) (Figure 1). At that moment, plants were in 5-L containers filled with standard nursery potting soil, without additional fertiliser. The experiment was carried out in greenhouse conditions. During the drought treatments, irrigation was withheld, whereas control plants were watered regularly by experienced greenhouse staff.

Following the spring treatment, drought-treated saplings were classified into two groups based on the developed visual drought stress symptoms: less and more than 50% of the leaves desiccated (D<50 and D>50) (Figure 1). The assessment of the number of leaves desiccated was performed in the recovery phase when it became visually clear which leaves had desiccated. The assessment was performed by a single observer. Severe leaf desiccation is tightly coupled with hydraulic failure, resulting in loss of leaf vascular conductance [59]. At the end of the second treatment, this resulted in 6 categories of plants: control–control (C-C, *n* = 71), control–drought (C-D, *n* = 67), drought with less than 50% of leaves desiccated–control (D<50-C, *n* = 46), drought with less than 50% of leaves desiccated–drought (D<50-D, *n* = 43), drought with more than 50% of leaves desiccated–control (D>50-C, *n* = 26) and drought with more than 50% of leaves desiccated–drought (D>50-D, *n* = 28) (Figure 1). Plants in the category D>50 resprouted after the first drought period and both categories D>50-C and D>50-D are further called the severe spring-drought categories. Plants in the categories C-D and D<50-D lost their foliage in the second drought period and did not resprout afterward (Figure 6). These two categories are further called the severe summer-drought categories. Plants in the category D>50-D had not yet developed any leaf desiccation symptoms when the second drought period ended. At the beginning of the growing season of 2023, only 7 plants had died off.

During the winter of 2022, plants remained in the greenhouse. In early spring of 2023, plants were transferred to a larger pot (7-L) and placed, randomly and intermixed, on a container field at the Research Institute of Nature and Forest in Geraardsbergen, Belgium. In the winters of 2023 and 2024, plants were transferred to larger pots, 10-L and 15-L, respectively. At each transfer, plants were individually and randomly intermingled on the container field. During the growing seasons plants were well watered by experienced technicians. Because of the common garden set-up, all plants were handled in the same way, except for the water-withholding treatments. Any disturbance of the root system by the repotting in the years after the drought treatments will have occurred in control plants as well as in treated plants. Therefore, we considered any influence by the repotting on the drought responses as insignificant.

### 4.2. Measurements and Observations

In 2023, 2024 and 2025, phenological observations of spring bud burst and autumn leaf senescence were performed using two scoring protocols following previous studies [28,57]. For bud burst, the scores were: 1, winter buds; 2, first green parts of the leaves protruding from the buds; 3, leaves in the process of unfolding; 4, leaves unfolded; 5, leaves enlarged and mature. For leaf senescence: 1, green leaves; 2, light green leaves; 3, yellowing leaves; 4, yellow leaves turning brown; 5, brown leaves, beginning to fall. Days of observation are indicated in Table 1.

During each growing season, leaf chlorophyll content index (using CCM-200, Opti-Sciences, Inc., Hudson, NH, USA) was measured at regular intervals on a representative, damage free and mature leaf on a representative short shoot in the centre of the young crown of each plant. These were always shaded leaves. The CCM-200 instrument measures optical absorbance at 653 nm (chlorophyll) and 931 nm (near infra-red). The relative chlorophyll content is the ratio of optical transmission at 931 nm to optical transmission at 653 nm [60]. While the chlorophyll content index (CCI) of a leaf, as measured by the CCM-200 instrument, is an effective, non-destructive proxy for leaf photosynthetic capacity, due to its coupling with maximum carboxylation rates [61], the accuracy of the measurements can be limited by optical saturation at high chlorophyll concentrations, as well as by specific leaf structural characteristics [62].

Each winter, height and diameter at 5 cm above soil level were measured. Annual increments of growth were calculated by subtracting height/diameter recorded in the preceding winter from the corresponding height/diameter measured in the given winter.

### 4.3. Statistical Analysis

All statistical analyses were performed with the open-source statistical software R version 4.5.1 [63]. Although the double controls and the different drought categories of saplings were not equal in number of plants, the applied modelling techniques can handle unbalanced datasets [64]. The phenological observations were modelled with cumulative logistic regression, whereas the chlorophyll content index measurements and the height and diameter increments were modelled applying linear regression. When a model involved repeated measurements on the same saplings (all phenological models and chlorophyll content index models), mixed-effects modelling was applied with a unique plant identifier included as a random effect (random intercept).

Cumulative logistic regression models (proportional odds models) were fitted separately for the two phenophases, spring bud burst and autumn leaf senescence, independently for each year (2023–2025, i.e., 6 models in total). For both phenophase, we modelled the cumulative probability p_k_ = P(Y ≤ k) that a plant had reached a maximum score level k on a given observation day with k, denoting the cumulative thresholds between the 5 ordinal score levels for the two phenophases. To facilitate intuitive visual interpretation in the figures of the modelled results, the direction of the ordinal scales was defined as follows. For spring bud burst, the ordinal scores were defined from score 5 (fully unfolded leaves) to score 1 (buds in winter rest), ensuring that cumulative probabilities (P(Y ≤ k)) yielded ascending curves over time. For autumn leaf senescence, ordinal scores were defined from 1 (green leaves) to 5 (fully senesced leaves starting to fall off), yielding descending curves over time. An interaction between the day of observation (Day) and drought treatment category in 2022 (Tre, categorical variable with 6 levels: C-C, C-D, D<50-C, D<50-D, D>50-C and D>50-D) allowed the phenological phase to develop quicker or slower in the different drought categories. Height of the plants (Hei), measured in the winter preceding the bud burst or measured in the winter following the leaf senescence in each observation year, was included as a covariate.

(1) logit(p_k_) = log(p_k_/(1 − p_k_)) = α_k −_ β_Day_Day − β_Tre_Tre − β_Day:Tre_ Day:Tre − β_Hei_Hei

Because of (quasi) separation, we fitted the model for bud burst in 2025 without the interaction term.

When appropriate, we calculated the duration of the phenophase bud burst as the number of days to develop from the transition between score 1 and 2 to the transition between score 4 and 5. This was calculated for each drought category (different levels in the categorical variable Tre) and for the mean height of the plants, using the following formula:

(2) Time lag = Day_5|4_ − Day_2|1_ = (α_5|4_ − α_2|1_)/(β_Day_ + β_Day:Tre_)

When appropriate, we calculated for the phenophase leaf senescence, the difference in days (Δ Day) between the double control (C-C) and the different drought categories (levels i in the variable Tre) for the transition from score 2 to score 3 and for the mean height of the plants (Hei), using the following formulas:

(3) Day_2|3,i_ = (α_2|3 −_ β_Tre,i_ − β_Hei_Hei)/(β_Day_ + β_Day:Tre,i_)

(4) Δ Day_2|3,i_ = Day_2|3,i_ − Day_2|3,C-C_

Linear regression models were applied for the chlorophyll content index measurements (Chl) for each year, from 2023 to 2025. Hei is the height at the winter preceding the analysed growth year. A polynomial of the second degree for the variable Day allowed the modelled chlorophyll content index to vary in a non-linear way between the three measurement time points (3 models), as the natural evolution of this physiological parameter during a growing season is unimodal.

(5) Chl = α + β_Day_Day + β_Day2_ Day^2^ + β_Tre_Tre + β_Day:Tre_Day:Tre + β_Day2:Tre_Day^2^:Tre + β_Hei_Hei

Linear regression models were fitted for height and diameter increments (Inc_Hei_ and Inc_Dia_) in each studied year (2023 till 2025). Hei or Dia is the height or diameter at the winter preceding the analysed growth year (6 models).

(6) Inc_Hei_ = α + β_Tre_Tre + β_Hei_Hei

(7) Inc_Dia_ = α + β_Tre_Tre + β_Dia_Dia

## 5. Conclusions

Our study demonstrates that drought legacy effects in European beech extend beyond the year of stress exposure and can persist across multiple growing seasons, affecting both phenology and growth recovery. Remarkably, we found evidence that legacy effects can re-emerge after having seemingly disappeared, suggesting that drought-induced stress memories may remain latent and become expressed under environmental conditions that allow their manifestation. The magnitude and expression of these legacy effects depended not only on drought severity and subsequent environmental conditions but also on the timing of the drought event within the growing season. Our findings further reveal a temporal decoupling between photosynthetic recovery and biomass accumulation, highlighting that restoration of physiological activity does not necessarily translate immediately into growth recovery and that growth may subsequently exceed control levels through compensatory responses. These delayed and context-dependent legacy effects should be considered when predicting forest responses to increasing drought frequency under climate change. Understanding how drought timing, severity, and environmental context shape long-term stress memory will become more important for assessing the resilience and adaptive capacity of temperate tree species.

This experimental study was not performed without limitations. The experiment was conducted under strongly controlled conditions in a common garden set-up, which allows for studying the effect of a single changing environmental factor on plant responses while holding all other variables constant. However, this high level of environmental control inherently simplifies natural conditions. In a natural forest ecosystem, multiple biotic and abiotic stress factors interact simultaneously, meaning that the physiological responses observed here cannot be directly extrapolated to complex, field-scale environments without caution. Our results urge future studies to include multi-site field experiments, integration of epigenetic markers and long-term carbon allocation tracking.

## Figures and Tables

**Table 1:** Days of observation for the phenophases bud burst and leaf senescence and of the measurements of leaf chlorophyll content index in the years 2023 till 2025.

Year	Bud Burst	Chlorophyll Content Index	Leaf Senescence
2023	18 and 24 April, 2 and 16 May	19 June, 1 August and 15 September	25 September and 30 October
2024	8, 15, 22 and 29 April	13 June, 2 August and 27 September	23 September, 15 October and 12 November
2025	17, 22 and 28 April	2 June, 28 July and 25 September	13 and 27 October

## Data Availability

The original data presented in the study are openly available in: 10.5281/zenodo.21852413.

## Supplementary Materials

The following supporting information can be downloaded at https://www.mdpi.com/article/10.3390/plants15182867/s1. Figure S1: Heat maps of continency tables of the bud burst scores in the springs of 2023, 2024 and 2025, and of the leaf senescence scores in the autumns of 2023, 2024 and 2025, according to the different drought categories. Drought categories are summarised in Figure 1. Figure S2: Mean and standard deviation of the chlorophyll content index in 2023, 2024 and 2025, according to the different drought categories. Drought categories are summarised in Figure 1. Figure S3: Box plot of the height and diameter increments in 2023, 2024 and 2025, according to the different drought categories. Drought categories are summarised in Figure 1. Table S1: Test statistics for the modelling of bud burst and leaf senescence in the years 2023 till 2025. The different drought categories (C-D, D<50-C, D<50-D, D>50-C and D>50-D) were compared to the standard double controls. For bud burst, plant height (Hei) at the beginning of the respective growing season was included as a covariate, whereas for leaf senescence, plant height at the end of the respective growing. Drought categories are summarised in Figure 1. Table S2: Test statistics for the modelling of bud burst and leaf senescence in the years 2023 till 2025. The different drought categories (C-D, D<50-C, D<50-D, D>50-C and D>50-D) were compared to the standard double controls. For bud burst, plant height (Hei) at the beginning of the respective growing season was included as a covariate, whereas for leaf senescence, plant height at the end of the respective growing. Drought categories are summarised in Figure 1. Table S3: Test statistics for the modelling of the height and diameter increments in the years 2023 till 2025. The different drought categories (C-D, D<50-C, D<50-D, D>50-C and D>50-D) were compared to the standard double controls. For height and diameter increment, plant height (Hei) and diameter (Dia) at the beginning of the growing season were included as covariates, respectively. Drought categories are summarised in Figure 1.
