# Supplementary material for: Re-Appearing Legacy Effect in Timing of Autumnal Leaf Senescence and Compensation Growth After Severe Drought in Fagus sylvatica L
Source: Plants (Basel). 2026 Sep 19;15(18):2867. doi: 10.3390/plants15182867 (PMC13610978; doi:10.3390/plants15182867)
Supplement: Supplementary file 1 [file plants-15-02867-s001.zip › plants-4536242-supplementary.pdf]

Supplementary material

Re-appearing legacy effect in timing of autumnal leaf senescence and compensation growth after severe drought in *Fagus sylvatica* L.

Kristine Vander Mijnsbrugge [1,*], Sofie Vanneste [1], Sharmila Majumder [1], Marc Schouppe [1], Stefaan Moreels [1], Sharon Moreels [1], Simeon Beeckman [1]

[1] Department for Forest Ecology and Management, Research Institute for Nature and Forest (INBO), Geraards-bergen 9500, Belgium; sofie.vanneste@gmail.com (S.V.); sharmilamou.126@gmail.com (S.Ma.); marc.schouppe@inbo.be (M.S.), stefaan.moreels@inbo.be (St.M.); sharon.moreels@inbo.be (S.Mo.); simeon.beeckman@gmail.com (S.B.)

* Correspondence: kristine.vandermijnsbrugge@inbo.be

1. Supplementary figures

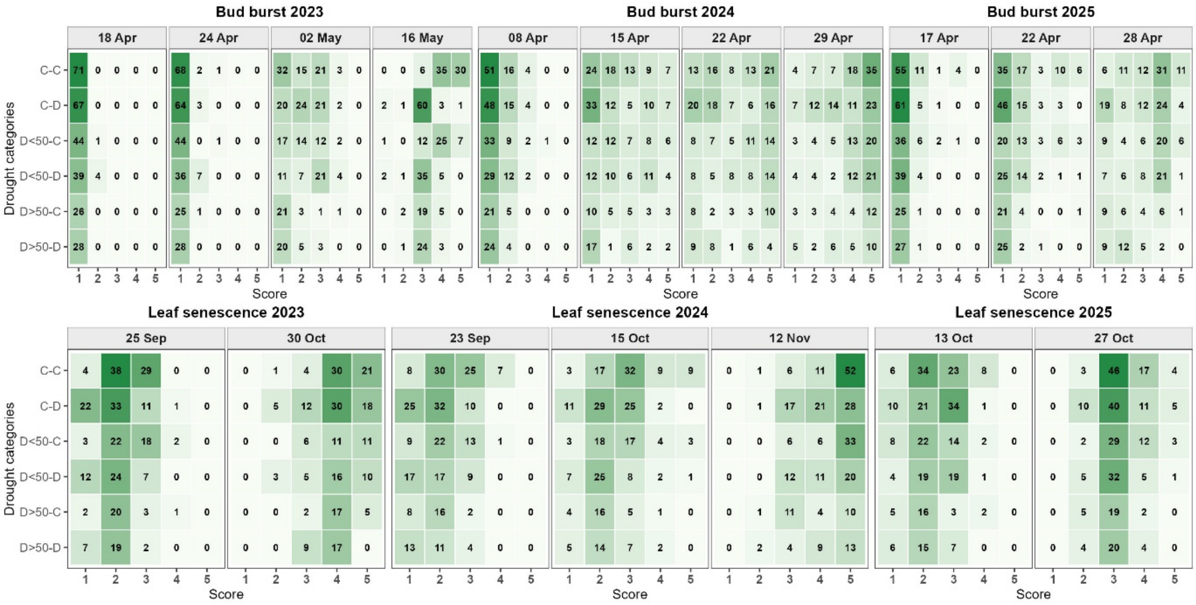

Figure S1. Heat maps of continency tables of the bud burst scores in the springs of 2023, 2024 and 2025, and of the leaf senescence scores in the autumns of 2023, 2024 and 2025, according to the different drought categories. Drought categories are summarised in Figure 1.

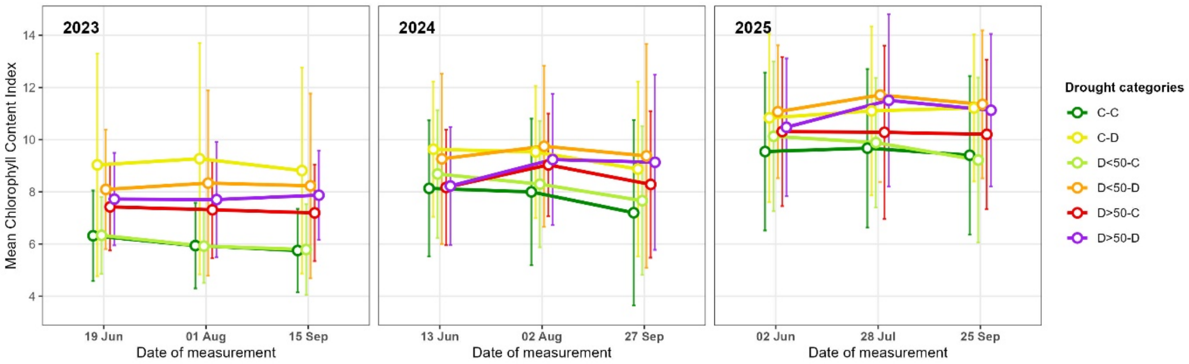

Figure S2. Mean and standard deviation of the chlorophyll content index in 2023, 2024 and 2025, according to the different drought categories. Drought categories are summarised in Figure 1.

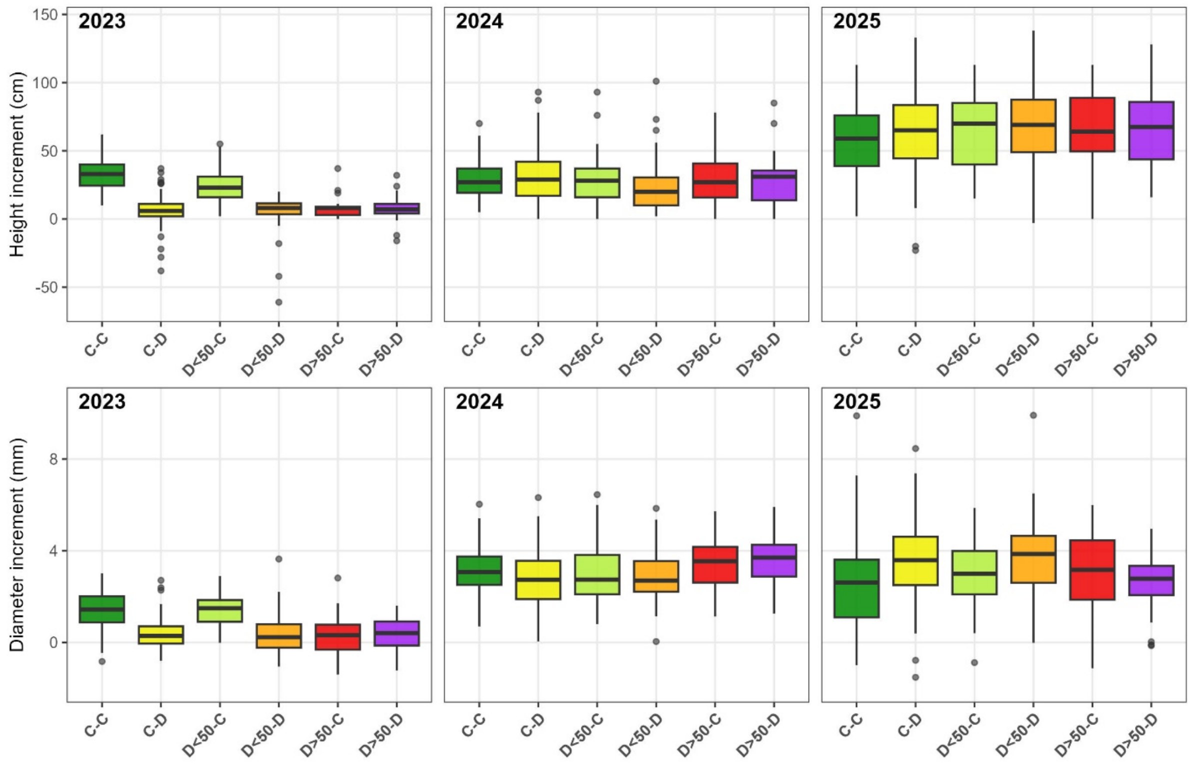

Figure S3. Box plot of the height and diameter increments in 2023, 2024 and 2025, according to the different drought categories. Drought categories are summarised in Figure 1.

2. Supplementary Tables

Table S1. Test statistics for the modelling of bud burst and leaf senescence in the years 2023 till 2025. The different drought categories (C-D, D<50-C, D<50-D, D>50-C and D>50-D) were compared to the standard double controls. For bud burst, plant height (Hei) at the beginning of the respective growing season was included as a covariate, whereas for leaf senescence, plant height at the end of the respective growing. Drought categories are summarised in Figure 1.

		bud burst				leaf senescence			
Year	Variable	Estimate	Std. error	z-value	*p*-value	Estimate	Std. error	z-value	*p*-value
2023	Day	-0.60	0.04	-14.51	<0.001***	0.24	0.03	9.24	<0.001***
	C-D	-4.15	0.73	-5.72	<0.001***	-0.84	0.66	-1.28	0.202
	D<50-C	-1.79	0.79	-2.25	0.024*	1.06	0.64	1.64	0.101
	D<50-D	-5.48	0.74	-7.41	<0.001***	-0.65	0.72	-0.90	0.369
	D>50-C	-0.94	1.17	-0.80	0.422	0.53	0.81	0.66	0.512
	D>50-D	-1.28	1.12	-1.14	0.253	-0.63	0.82	-0.76	0.445
	Hei22/Hei23	-0.01	0.01	-1.49	0.137	0.05	0.01	5	<0.001***
	C-D : Day	0.30	0.04	8.28	<0.001***	0.03	0.02	1.8	0.072
	D<50-C : Day	0.12	0.04	3.36	<0.001***	-0.01	0.02	-0.40	0.693
	D<50-D : Day	0.34	0.04	9.18	<0.001***	0.04	0.02	2.01	0.044*
	D>50-C : Day	0.19	0.05	3.70	<0.001***	0.01	0.02	0.66	0.508
	D>50-D : Day	0.20	0.05	4.09	<0.001***	0.00	0.02	-0.11	0.913
2024	Day	-0.81	0.06	-14.49	<0.001***	0.13	0.01	13.15	<0.001***
	C-D	0.86	1.70	0.50	0.615	-2.74	0.51	-5.43	<0.001***
	D<50-C	-1.27	1.64	-0.77	0.440	-1.31	0.52	-2.54	0.011*
	D<50-D	-1.14	1.85	-0.62	0.537	-2.84	0.58	-4.91	<0.001***
	D>50-C	2.48	2.19	1.13	0.258	-2.83	0.66	-4.30	<0.001***
	D>50-D	4.29	2.24	1.92	0.055	-3.26	0.66	-4.92	<0.001***
	Hei23/Hei24	0.03	0.23	0.12	0.903	-0.01	0.01	-1.06	0.291
	C-D : Day	0.11	0.05	2.33	0.020*	0.02	0.01	1.59	0.113
	D<50-C : Day	0.06	0.05	1.32	0.187	0.02	0.01	1.63	0.103
	D<50-D : Day	0.07	0.05	1.44	0.149	0.02	0.01	1.48	0.138
	D>50-C : Day	-0.09	0.07	-1.30	0.192	0.00	0.01	0.23	0.817
	D>50-D : Day	-0.04	0.07	-0.57	0.568	0.03	0.01	1.97	0.049*
2025	Day	-1.29	0.10	-13.29	<0.001***	0.27	0.03	8.06	<0.001***
	C-D	4.06	1.29	3.15	0.002**	-0.31	0.52	-0.59	0.556
	D<50-C	1.00	1.30	0.77	0.439	-1.04	0.56	-1.85	0.065
	D<50-D	2.39	1.36	1.77	0.078	-0.30	0.58	-0.52	0.605
	D>50-C	6.14	1.83	3.36	<0.001***	-1.67	0.69	-2.42	0.015*

D>50-D	7.58	1.84	4.12	<0.001***	-1.85	0.68	-2.74	0.006**
Hei24/Hei25	-0.01	0.00	-1.16	0.246	0.003	0.004	-0.91	0.364
C-D : Day					-0.03	0.04	-0.75	0.451
D<50-C : Day					0.07	0.04	1.64	0.101
D<50-D : Day					-0.06	0.05	-1.40	0.162
D>50-C : Day					-0.01	0.05	-0.27	0.784
D>50-D : Day					0.04	0.05	0.67	0.500

*** $p < 0.001$; ** $p < 0.01$; * $p < 0.05$

Table S2. Test statistics for the modelling of bud burst and leaf senescence in the years 2023 till 2025. The different drought categories (C-D, D<50-C, D<50-D, D>50-C and D>50-D) were compared to the standard double controls. For bud burst, plant height (Hei) at the beginning of the respective growing season was included as a covariate, whereas for leaf senescence, plant height at the end of the respective growing. Drought categories are summarised in Figure 1.

Year	Variable	Value	Std. error	DF	t-value	*p*-value
2023	(Intercept)	11.09	0.74	548	14.98	<0.001***
	C-D	2.49	0.41	274	6.07	<0.001***
	D<50-C	-0.32	0.45	274	-0.71	0.481
	D<50-D	1.73	0.46	274	3.75	<0.001***
	D>50-C	0.64	0.55	274	1.17	0.245
	D>50-D	0.82	0.54	274	1.51	0.131
	Day	-6.67	2.60	548	-2.57	0.011*
	Day^2	1.42	2.60	548	0.55	0.584
	Hei22	-0.06	0.01	274	-7.42	<0.001***
	C-D : Day	2.34	3.74	548	0.63	0.531
	D<50-C : Day	0.27	4.14	548	0.07	0.948
	D<50-D : Day	5.16	4.25	548	1.21	0.225
	D>50-C : Day	3.94	5.01	548	0.79	0.433
	D>50-D : Day	8.47	4.88	548	1.74	0.083
	C-D : Day^2	-5.11	3.73	548	-1.37	0.172
	D<50-C : Day^2	0.63	4.15	548	0.15	0.879
	D<50-D : Day^2	-1.94	4.24	548	-0.46	0.647
	D>50-C : Day^2	-1.42	5.02	548	-0.28	0.777
	D>50-D : Day^2	-0.19	4.89	548	-0.04	0.969
2024	(Intercept)	11.77	0.99	545	11.92	<0.001***
	C-D	0.35	0.53	272	0.67	0.505
	D<50-C	-0.04	0.51	272	-0.08	0.939
	D<50-D	0.44	0.58	272	0.76	0.447

	D>50-C	-0.47	0.66	272	-0.72	0.475
	D>50-D	-0.27	0.66	272	-0.41	0.682
	Day	-10.99	2.84	545	-3.87	<0.001***
	Day^2	-3.94	2.83	545	-1.39	0.165
	Hei23	-0.03	0.01	272	-4.25	<0.001***
	C-D : Day	1.54	4.08	545	0.38	0.706
	D<50-C : Day	-2.25	4.54	545	-0.50	0.621
	D<50-D : Day	12.06	4.61	545	2.62	0.009**
	D>50-C : Day	11.88	5.46	545	2.18	0.030*
	D>50-D : Day	21.36	5.32	545	4.02	<0.001***
	C-D : Day^2	1.31	4.07	545	0.32	0.748
	D<50-C : Day^2	2.41	4.54	545	0.53	0.596
	D<50-D : Day^2	-1.92	4.60	545	-0.42	0.677
	D>50-C : Day^2	-7.10	5.46	545	-1.30	0.194
	D>50-D : Day^2	-4.14	5.32	545	-0.78	0.436
2025	(Intercept)	13.10	0.95	544	13.76	<0.001***
	C-D	0.70	0.48	271	1.45	0.147
	D<50-C	-0.26	0.49	271	-0.53	0.596
	D<50-D	0.86	0.55	271	1.56	0.120
	D>50-C	-0.17	0.62	271	-0.27	0.790
	D>50-D	0.52	0.62	271	0.84	0.399
	Day	-1.20	3.65	544	-0.33	0.742
	Day^2	-2.50	3.65	544	-0.69	0.494
	Hei24	-0.02	0.01	271	-3.94	<0.001***
	C-D : Day	5.66	5.22	544	1.08	0.279
	D<50-C : Day	-10.00	5.78	544	-1.73	0.084
	D<50-D : Day	4.45	5.90	544	0.75	0.451
	D>50-C : Day	-0.07	6.98	544	-0.01	0.992
	D>50-D : Day	8.81	6.80	544	1.30	0.196
	C-D : Day^2	1.40	5.22	544	0.27	0.788
	D<50-C : Day^2	0.66	5.80	544	0.11	0.910
	D<50-D : Day^2	-4.32	5.89	544	-0.73	0.464
	D>50-C : Day^2	2.20	6.97	544	0.32	0.753
	D>50-D : Day^2	-7.17	6.79	544	-1.06	0.291

*** $p < 0.001$; ** $p < 0.01$; * $p < 0.05$

Table S3. Test statistics for the modelling of the height and diameter increments in the years 2023 till 2025. The different drought categories (C-D, D<50-C, D<50-D, D>50-C and D>50-D) were compared to the standard double controls. For height and diameter increment, plant height (Hei) and diameter (Dia) at the beginning of the growing season were included as covariates, respectively. Drought categories are summarised in Figure 1.

		Height increment				Diameter increment			
Year	Variable	Estimate	Std. error	t-value	*p*-value	Estimate	Std. error	t-value	*p*-value
2023	(Intercept)	40.39	3.71	10.89	<0.001***	3.58	0.44	8.05	<0.001***
	tr5C-D	-27.68	2.06	-13.42	<0.001***	-0.92	0.13	-7.10	<0.001***
	tr5D<50-C	-9.96	2.24	-4.46	<0.001***	-0.04	0.14	-0.25	0.805
	tr5D<50-D	-29.15	2.31	-12.64	<0.001***	-1.03	0.15	-7.04	<0.001***
	tr5D>50-C	-25.99	2.73	-9.51	<0.001***	-0.88	0.18	-4.96	<0.001***
	tr5D>50-D	-26.71	2.70	-9.89	<0.001***	-0.96	0.17	-5.59	<0.001***
	Hei22/Dia22	-0.08	0.04	-2.10	0.036*	-0.18	0.04	-5.02	<0.001***
2024	(Intercept)	44.54	6.96	6.40	<0.001***	2.47	0.73	3.36	<0.001***
	tr5C-D	-2.38	3.76	-0.63	0.527	-0.23	0.20	-1.18	0.238
	tr5D<50-C	-2.56	3.58	-0.71	0.476	-0.13	0.22	-0.62	0.537
	tr5D<50-D	-8.41	4.12	-2.04	0.042*	-0.13	0.23	-0.55	0.586
	tr5D>50-C	-5.89	4.66	-1.26	0.208	0.33	0.26	1.27	0.204
	tr5D>50-D	-4.89	4.69	-1.04	0.299	0.43	0.25	1.70	0.091
	Hei23/Dia23	-0.12	0.05	-2.34	0.020*	0.05	0.05	0.92	0.359
2025	(Intercept)	49.39	10.75	4.60	<0.001***	5.25	1.06	4.96	<0.001***
	tr5C-D	6.91	5.43	1.27	0.205	0.71	0.32	2.24	0.026*
	tr5D<50-C	9.35	5.53	1.69	0.092	0.15	0.35	0.44	0.657
	tr5D<50-D	15.10	6.21	2.43	0.016*	1.01	0.36	2.80	0.006**
	tr5D>50-C	10.34	7.05	1.47	0.143	0.63	0.42	1.52	0.130
	tr5D>50-D	11.37	6.98	1.63	0.104	-0.02	0.41	-0.06	0.956
	Hei24/Dia24	0.04	0.07	0.67	0.501	-0.16	0.06	-2.51	0.013*

*** $p < 0.001$; ** $p < 0.01$; * $p < 0.05$
